# The repositioning of hospitalized patients with reduced mobility: a prospective study

**DOI:** 10.1002/nop2.20

**Published:** 2015-07-14

**Authors:** Sharon Latimer, Wendy Chaboyer, Brigid M. Gillespie

**Affiliations:** ^1^ Griffith UniversitySchool of Nursing and MidwiferyMeadowbrookQueenslandAustralia; ^2^ Griffith UniversityNHMRC Research Centre for Excellence in Nursing Interventions (NCREN)Gold CoastQueenslandAustralia

**Keywords:** Nursing practice, nursing shift, predictors, pressure injuries, repositioning

## Abstract

**Aim:**

To determine the frequency of patient repositioning across three consecutive nursing shifts (day, evening and night) and to identify predictors of repositioning frequency.

**Background:**

Patient repositioning is a frequently implemented pressure injury prevention strategy. Yet, little is known about how often it should be implemented, or the frequency of movement among hospitalized patients with reduced mobility.

**Design:**

An observational prospective study.

**Methods:**

Chart audits were used to gather clinical and demographic data. Semi‐structured observations were conducted every 30 minutes for a continuous 24‐hour period. Observational data included the patient's body position, the frequency of repositioning, assistance require to reposition and the use of support surfaces.

**Results:**

Patients were repositioned frequently during the day and evening and least at night time. Elevation of the head of the bed (1–45°) was the most frequently adopted position. The independent predictors of repositioning frequency were age and gender, with older patients and males repositioned less frequently.

## Introduction

Pressure injuries (PI) or pressure ulcers are a significant patient safety and quality healthcare issue (Moore *et al*. 2011); many of which are avoidable (Black *et al*. 2011). A range of prevention strategies is recommended in clinical practice guidelines (CPG) for the pressure injury prevention (PIP), with repositioning a core component (NPUAP/EPUAP/PPPIA 2014). Current evidence into the effectiveness and timing of repositioning as a PIP strategy is not only limited, but also lacks consensus (Miles *et al*. 2013, Gillespie *et al*. 2014). Furthermore, little is known about the body positions of hospital patients (Chaboyer *et al*. 2013). Because of this gap in understanding, a study was undertaken to describe the repositioning of medical patients over a continuous 24‐hour period.

### Background

PI are an adverse event caused by the mechanical factors of pressure, shearing and/or friction, resulting in localized damage to either the skin and/or underlying tissue (Coleman *et al*. 2014, NPUAP/EPUAP/PPPIA 2014). Internationally, the prevalence of hospital acquired pressure injuries (HAPI) varies with the most recent figures ranging from 7·4% in Australia (Mulligan *et al*. 2011), 12·3% in the US (VanGilder *et al*. 2009) to 25% in Sweden (Moore *et al*. 2013). PI have negative impacts for patients, healthcare staff and organizations such as emotional and physical distress (Latimer *et al*. 2014) and increased workload (Moore & Price 2004, Chaboyer & Gillespie 2014). For the period in 2012–2013, it was estimated to cost A$983 million per annum to treatment all of the PI in Australian public hospitals (Nguyen *et al*. 2014). As a result, the prevention and management of PI is a priority healthcare area (Institute of Medicine 2012, National Health Service Commissioning Board 2013, Australian Commission on Safety and Quality in Health Care 2012).

Current CPG outline several PIP strategies, including PI risk assessment, PIP management plan, the appropriate use of support surfaces, continence management, patient education, skin protection, nutritional assessment, adequate nutrition and regular repositioning (NPUAP/EPUAP/PPPIA 2014). While the assessment of a patient's PI risk is the first step in prevention (NPUAP/EPUAP/PPPIA 2014), repositioning is viewed as a cornerstone PIP strategy (Moore & Cowman 2012, NPUAP/EPUAP/PPPIA 2014). Repositioning is defined as the movement of patients from one position to another in an effort to alleviate or redistribute any pressure exerted on the body tissues (Gillespie *et al*. 2014).

There are significant complexities around how repositioning is used to prevent PI. For example, many nurses implement 2‐4 hourly repositioning for PIP (Miles *et al*. 2013), although there is little evidence supporting this practice (Young 2004, Moore *et al*. 2011, Coleman *et al*. 2014). Furthermore, PIP researchers and clinical experts are yet to agree on the ideal frequency of repositioning to prevent PI, adding to this uncertainty (Miles *et al*. 2013, Gillespie *et al*. 2014). Compounding this is the patient's contribution to repositioning through their own movement. For example, one study found more than half of participants changed their position in between the scheduled repositioning by nurses (Young 2004), with many patients adopting positions that increase their PI risk (McInnes *et al*. 2013).

During hospitalization, patients are placed in, or adopt several body positions including supine, degree of tilt (left or right lateral), head of bed elevation (HOBE) between 1‐90°, sitting or walking (Moore *et al*. 2011, McInnes *et al*. 2013). The use of a 30° of tilt to reduce PI has been found to reduce PI incidence compared with usual PIP care (Moore *et al*. 2011). This position is achieved by rolling the patient laterally to a slightly tilted 30° position and supported in this position by pillows (Moore *et al*. 2011). When patients are in bed, the HOBE or the angle the bed head is raised, can increase the pressure placed on the coccyx, placing them at greater PI risk (Wilson 2008, Moore *et al*. 2011). CPG recommend HOBE should be maintained at, or below 30°, or the lowest elevation congruent to the management of the patient's medical condition (NPUAP/EPUAP/PPPIA 2014). A recent observational study reported participants often adopted positions that placed them at an increased risk of PI, with a HOBE ranging between 1‐90° (Chaboyer *et al*. 2013, McInnes *et al*. 2013). Our study has three aims; first to determine the patient's body position and frequency of repositioning among hospitalized patients with reduced mobility, across three consecutive nursing shifts (day, evening and night), second, to determine if there is a difference in the repositioning frequency across the three nursing shifts; finally, to identify factors that predict repositioning frequency.

## Method

### Design

**Figure 1:**
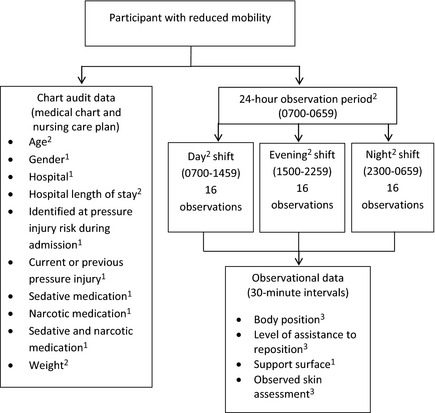
Study and data collection overview. 1: Dichotomous measure; 2: Continuous measure; 3: Categorical measure.

This observational study incorporated two data collection methods: chart audits and semi‐structured observations. Figure 1 depicts an overview of the study, data collection methods and the predictors selected for testing.

Previous PIP research (Defloor *et al*. 2005, Nixon *et al*. 2007, Brown *et al*. 2009, Coleman *et al*. 2013, McInnes *et al*. 2013, Miles *et al*. 2013) and the clinical judgement of the research team informed the selection of the 12 predictor variables for this study (age, gender, hospital, hospital length of stay (HLOS) at data collection, identified at PI risk during admission, sedative medication, narcotic medication, sedative and narcotic medication, weight, previous or current PI, observed skin assessment, support surface).

### Participant recruitment

Using a consecutive sampling plan, participants were recruited from four medical units located at two large Australian hospitals (Hospital A: 986 beds and Hospital B:450 beds) (Queensland Health 2012). Both are public hospitals, offering a wide range of inpatient and specialist clinical services (Queensland Health 2012). All aspects of patient care, including repositioning, were planned by the Registered Nurse (RN) and implemented by either the RN, Endorsed Enrolled Nurses, Enrolled Nurses or Assistants in Nursing. The study inclusion criteria were: aged ≥18 years; a HLOS at time of recruitment of ≥3 days; ability to provide an informed consent; and reduced mobility (equipment and/or staff needed to ambulate/reposition in bed). During recruitment, verbal and written overviews of the study were provided to potential participants. Written consent was obtained from those willing to participate. Individuals could only be recruited once to the study. To increase model stability and generalizability and to increase the robustness of the regression analysis, we aimed to recruit 240 participants, or a minimum of 20 participants per predictor as recommended (Polit 2010, Field 2013).

### Data collection

Data collection was undertaken by the research team on 28 randomly selected days from November 2011–February 2012. Seven days were spent in each of the four medical units; totalling 28 days. The sequence of data collection days was randomly selected using computer software to ensure that data collection occurred in each medical unit, on each day of the week. Development of the data collection tools were informed by current CPG for PIP (NPUAP/EPUAP/PPPIA 2014) and the results of previous research (Defloor *et al*. 2005, Webster *et al*. 2011). The data collection methods and level of measurement of the variables are outlined in Figure 1. The following data were collected from the medical chart: patient age and gender, hospital, HLOS at data collection, diagnosis, comorbidities, identified at pressure injury risk during admission and current or previous PI. Pressure injury risk assessment, sedative medication, narcotic medication and patient weight data were collected from the nursing care plan.

The research team collected semi‐structured observational data (Figure 1) at 30‐minute intervals over a continuous 24‐hour period (0700‐0659). These observational data were collected across three data collection periods coinciding with the scheduled nursing shift periods (i.e. 0700‐1459; 1500‐2259 and 2300‐0659). There were 16 separate observation points per shift, totalling 48 observations per participant in a 24‐hour period (Figure 1).

Observational data collected on the participant's body position included supine, HOBE, lateral (left or right), sitting, walking or unable to observe. Participants observed with a HOBE also had the degree of elevation (1‐90°) recorded. There were times when participants could not be observed, for example, during showering or having procedures in another department. When this occurred, ‘unable to observe’ was recorded at the data collection point. The level of observed assistance provided to reposition participants was also documented in terms of participant independently repositioned, with human (staff/family) assistance, with the use of equipment, or not observed.

In this study, repositioning was defined as any observed body position change, which results in pressure alleviation or redistribution in a body part (McInnes *et al*. 2013, Gillespie *et al*. 2014). Repositioning ranges from small positional shifts executed independently by the patient, to repositioning performed by the healthcare staff on behalf of the patient (Wilson 2008, Moore & Cowman 2012), with or without equipment such as a slide sheet or hoist (McInnes *et al*. 2013). At each 30‐minute interval, the participants’ body position was noted, along with any observed assistance/equipment. For example, repositioning occurred if the participant's position was observed to change from supine to HOBE of 45°. If the participant then remained at a HOBE of 45° for the next three observation periods (one and a half hours), then HOBE 45° was recorded, but no change to their body position was deemed to have occurred. The four repositioning outcome variables (frequency of all repositioning, day, evening, night shift observations) were continuous.

The implementation of support surfaces included one or more of the following: pressure relieving mattress, seating cushions, foam wedges, pillows, sheepskins, bandages used to protect limbs from friction. The visible areas of the participant's skin (usually arms and lower legs) were observed and recorded as being healthy, clammy, discoloured, thin and frail, dry or a combination, such as dry and discoloured.

The reliability of the data collection tools was tested through the conduction of a pre and post pilot study. Inter‐rater testing was also used to test the consensus between the research team involved in data collection. In the pre‐pilot study, chart and semi‐structured observational data were collected on five participants, with a 92% inter‐rater reliability achieved. Following this, minor tool adjustments were made (e.g. adding ‘not applicable’ to some questions). A subsequent post pilot study conducted on five different participants was undertaken, yielding a 96% intra‐rater reliability result.

Prior to the commencement of the data collection, ethics approval was granted by the Human Research Ethics Committees at both hospitals (HREC/11/QTHS/111) and the university (NRS/40/11/HREC).

### Data analysis

The data were entered into the IBM SPSS statistics software (version 22·0, Chicago, IL, USA, 2013), then cleaned and checked for accuracy. Univariate analysis was undertaken, with level of distribution of the continuous data checked. All dichotomous predictor variables were dummy coded (i.e. 0, 1). The assumptions for the statistical tests of a one way repeated measures analysis of variance (anova) and multiple regression were checked. For the multiple regression analysis, a model building approach was used. Simple linear univariate analysis was first undertaken for the continuous outcome variable, frequency of repositioning and the 12 predictor variables. Following this, all statistically significant predictors (*P *<* *0·05) were simultaneously entered into a multiple regression analysis for the outcome variable, frequency of repositioning.

## Results

Table 1 outlines the demographic and clinical characteristics of the participants. The sample (*n *=* *241) consisted of more males than females and the median age was 70 years. Almost two‐fifths (*n *=* *94; 39·0%) of participants aged 55 years and older were identified at PI risk during their hospitalization. Sedatives or narcotics were prescribed for half of the participants.

**Table 1:** Demographics of sample (*n *=* *241)

Demographic/clinical characteristic	*n* (%)	Range	Md (IQR)
Age (years)		18‐94	70·0 (57·0‐80·0)
Male	142 (58·9)		
Hospital			
A	165 (68·5)		
B	76 (31·5)		
Hospital length of stay at data collection (days)		3‐110	5 (3‐8)
Number of comobidities		0‐11	2 (2‐5)
Weight (kg)		43‐158	76·3 (60·0‐90·3)
Medications			
Sedatives	50 (20·7)		
Narcotics	70 (29·0)		
Sedatives and narcotics	17 (7·1)		
Identified at pressure injury risk during hospitalisation	121 (50·2)		
Observed skin assessment category			
More than one category	167 (69·3)		
Healthy	51 (21·2)		
Thin and frail	13 (5·4)		
Dry	6 (2·5)		
Not observed	4 (1·7)		
Current or previous pressure injury	20 (8·3)		

Table 2 contains the frequency of the observed participants’ body position data. Participants were mostly observed in or around their hospital bed, with very few seen walking. Across the three shift periods, HOBE 1‐45° was the most frequently adopted body position by participants. More than two‐thirds of participants (*n *=* *160; 66·4%) were able to move themselves in bed, either independently or with the assistance of bed mechanics such as a grab rail. When mobilizing out‐of‐bed, almost half of participants (*n *=* *111; 46·1%) used multiple mobility strategies such as walking sticks and wheelchairs.

**Table 2:** Frequency of observed participant body position by shift (*n *=* *241)

Participant body position	AM *n* (%)	PM *n* (%)	ND *n* (%)
HOBE^a^ 1–45°	1099 (28·6%)	1282 (33·3%)	1428 (37·0%)
HOBE^a^ 46–90°	360 (9·4%)	346 (9·0%)	158 (4·1%)
Sitting	1231 (32·0%)	670 (17·4%)	191 (5·0%)
Supine	103 (2·7%)	162 (4·2%)	187 (4·8%)
Walking	196 (5·1%)	114 (3·0%)	64 (1·7)
Left Lateral	143 (3·7%)	273 (7·1%)	662 (17·2%)
Right Lateral	131 (3·4%)	342 (8·9%)	700 (18·2%)
Unable to observe	580 (15·1%)	657 (17·1%)	466 (12·0%)

a Head of bed elevation, ° Degrees.

The results of the one way repeated measures analysis of variance (anova) on the difference in the repositioning frequency across the three nursing shifts are presented in Table 3. Participants were observed to be repositioned on average 15 times (M 14·5; sd 6·9) over a 24‐hour period; 0·6 times an hour or the equivalent of once every 1·7 hours. Participants were repositioning on average 0·7, 0·6 and 0·5 times per hour on the respective day, evening and night shift. Put differently, on average participants were repositioned once every 1·4 hours on day shift, once every 1·7 hours on evening shift and once every 2 hours on night shift. Repositioning was similar during the day and evening shift, with fewer average repositionings observed at night. The Mauchly's test results indicate a violation of the assumption of sphericity (χ^2^ = 14·10; d.f. 2; *P *=* *0·001), consequently Greenhouse–Geisser corrected tests are reported (197·09; d.f. 1·89; *F* = 16·09; *P *<0 .001). These results show there was a difference in the repositioning frequency across three shift periods (Wilks’ Lambda 0·89, *F* (2,239) = 14·09; *P *<* *0·001; multivariate partial squared = 0·105). The posthoc analysis shows participants were repositioned less frequently on the night shift compared with the morning (*P *<* *0·001) and afternoon (*P *<* *0·001) shifts.

**Table 3:** Difference in the repositioning frequencies between shifts

Shift	*N*	M	sd	F	CI 95%		*P* value
					Lower	Upper	
Day shift (0700‐1459)	241	5·6	2·7	14·09	5·239	5·931	<0·001
Evening shift (1500‐2259)	241	5·1	3·0		4·703	5·463	
Night shift (2300‐0659)	241	4·3	3·3		3·893	4·738	

Univariate analysis of the outcome variable frequency of participant repositioning and the 12 predictor variables showed two predictors were statistically significant: age (ß* *=* *−0·054, 95% CI = −0·104‐0·003; *P *=* *0·038) and gender (ß = −2·066, 95% CI = −3·830 to −0·302; *P *=* *0·022). These two significant predictors were inserted into a multiple regression model using the simultaneous method. The results yielded an *R* square (*R* ^2^) 0·038 (Adjusted *R* ^2^ = 0·030; *F* = 4·663; *P *=* *0·010) suggesting the predictive ability of the model, albeit significant, accounted for a small 3·8% of the variance. Both predictor variables were statistically significant: age (ß* *=* *−0·051, 95% CI = −0·101 to 0·000; *P *=* *0·048) and gender (ß* *=* *−1·97, 95% CI = −3·729 to −0·217; *P *=* *0·028). Our results show that as the participants’ age increased, there was a decrease in the frequency of their repositioning. We also found females were more frequently repositioned compared with males. Multicollinearity between the predictor variables was checked, with none evident (tolerance 0·997; variance inflation factor 1·003).

## Discussion

### Frequency of repositioning

In this study, we found participants with reduced mobility were repositioned more frequently than might have been previously believed, with participants moving once every 1·7 hours. Our finding supports a growing body of literature that indicates hospital patients are moving (Chaboyer *et al*. 2013, McInnes *et al*. 2013). Yet, although patient repositioning is occurring frequently, evidence suggests bed‐ridden patients receiving a 2‐hourly repositioning schedule and those with restricted movement, remain at high risk for PI (Peterson *et al*. 2013). This could be because the repositioning techniques do not sufficiently relieve the pressure experienced by the skin and underlying tissue (Peterson *et al*. 2013). This current study did not investigate this issue. Little robust evidence exists to support the timing of the frequency of repositioning to prevent PI (Gillespie *et al*. 2014). Adopting a targeted and individualized approach to repositioning (Sprigle & Sonenblum 2011, NPUAP/EPUAP/PPPIA 2014, Chaboyer *et al*. 2015) is recommended as a way to better meet patients’ PIP needs. This can only be achieved with organizational support in terms of human and equipment resource provision (Gunningberg 2005).

### Participant's body position

Repositioning positions for PIP include supine, degree of tilt (left or right lateral), HOBE, sitting and walking (NPUAP/EPUAP/PPPIA 2014). Across all three shift periods (day, evening and night) participants in our study favoured the HOBE position of 1‐45°. Other recent studies have reported similar findings, with patients preferring a HOBE position between 1‐90° (Chaboyer *et al*. 2013, McInnes *et al*. 2013). A HOBE of 30° or greater, places the patient at greater risk of PI due to the increased friction and loading placed on the buttocks and sacrum (Wilson 2008, McInnes *et al*. 2013), with current CPG recommending a degree of tilt of less than 30° be used to prevent PI (NPUAP/EPUAP/PPPIA 2014). In addition, previous repositioning studies report participants changed their positions independently, from the scheduled repositioning regime (Young 2004), with many patients placing themselves in a position that increased their PI risk (McInnes *et al*. 2013). Several studies have reported hospitalized patients spend a significant amount of time lying on their bed and very little time walking around the ward, despite their ability to do so (Brown *et al*. 2009, Kuys *et al*. 2012, McInnes *et al*. 2013). Our study had similar findings. There is a need for nurses to educate patients about PIP and prompt them to adopt body positions that reduce their PI risk.

Hospitalized patients experience several barriers to regular repositioning including the presence of medical equipment (e.g. in‐dwelling catheters or intravenous lines), pain and weakness and a lack of staff availability to assist in ambulation (Brown *et al*. 2007). Encouraging patients to intermittently dangle their legs over the edge of the bed is an activity many patients can undertake independently. It has demonstrated benefits such as increased postoperative mobilization and improved pain management (Morris *et al*. 2010) and encourages patients to participate in their care (Chaboyer & Gillespie 2014, Latimer *et al*. 2014).

### Difference in the frequency of repositioning across three nursing shifts

Our results show the frequency of participants repositioning declined from the morning to evening shift and then the night shift. Participants in our study were repositioned less frequently during the night shift compared with the morning and evening shift. These results mirror recent similar studies (Brown *et al*. 2009, Chaboyer *et al*. 2013, McInnes *et al*. 2013). There may be several reasons for reduced repositioning during night shift including the presence of less nursing staff to implement the strategy (Gillespie *et al*. 2014), the importance of rest and sleep in the healing process (Humphries 2008) and the influence of sedative and narcotic medication (Nijs *et al*. 2009, Gillespie *et al*. 2014). Our results confirm patients are not being repositioned as frequently as they are during the day, which may place them at greater risk of PI. However, it may be that during the night shift, nurses are using their clinical judgement to assess the patient's PIP requirements (Webster *et al*. 2011); determining a reduced need for repositioning.

### Predictors of the frequency of repositioning

Our study found that as the participant's age increased, the frequency of their repositioning decreased. This finding is of concern, given that the majority of participants who were identified at risk for PI were aged 55 years and older. Our results contrast those of a study into various repositioning schedules (2‐6 hourly experimental repositioning and standard care), which found there were no differences in the care received based on the patient's age (Defloor *et al*. 2005). This difference in results may be due to the large differences in the sample sizes between the studies.

Nursing or patient factors could explain our results. Numerous studies have reported varied implementation of CPG by nurses (Sving *et al*. 2012, Moore 2013) including repositioning (Gunningberg 2005, Vanderwee *et al*. 2007). Two European studies report the majority of patients at risk of PI were not regularly repositioned (Gunningberg 2005, Vanderwee *et al*. 2007), suggesting that PIP strategies were either not appropriately allocated (Vanderwee *et al*. 2007) or were aimed at the bed‐ridden patient (Gunningberg 2005). Several reasons may explain a lack of repositioning. Poor care planning has been identified as a contributing factor, with 70% of patients in one study, not having regular repositioning documented in their care plan (Leach 2008). It has also been suggested nurses’ poor attitude towards PI and not their PIP knowledge, significantly correlated with the poor delivery of PIP care (Demarré *et al*. 2012). Recent studies found nurses know about PIP and encouraging patient participation (Ilesanmi *et al*. 2012), however, it appears some nurses need support to implement these strategies into their clinical practice (Chaboyer & Gillespie 2014, Chaboyer *et al*. 2015).

Patient factors could also contribute to our findings. It could be that patients refused to be repositioned, because they were in a comfortable resting position; however, these data were not collected. It has also been found that many hospitalized patients are physically and psychologically inactive; spending a great deal of time in their bed doing little or nothing (Kuys *et al*. 2012). For many, their physical inactivity was not related to their ability to mobilize (Brown *et al*. 2009, Kuys *et al*. 2012, McInnes *et al*. 2013). Older hospitalized patients experience a fear of falling which significantly reduces their mobility and functional ability (Boltz *et al*. 2014). Depression in older adults has also been found to increase their risk of falls (Turcu *et al*. 2004). Coupled with this, frailty in older patients has also been associated with depression, increased levels of anxiety and a reduced sense of control (Dent & Hoogendijk 2014). While these factors could explain our findings, it may be simply that the patient's bed is the most comfortable place for them (Brown *et al*. 2007). Patient education about PIP and patient participation in their care, should occur during the initial nursing PI risk assessment and continue throughout the patient's hospitalization.

Gender differences in the delivery of nursing care have been reported in the areas of cardiology (Poisson *et al*. 2010) and medication errors (Latimer *et al*. 2011). Our study found male participants were less frequently repositioned compared with females. We also found more males were identified at PI risk than were females. Our findings contradict Defloor *et al*. (2005) who reported that gender was not a significant factor in repositioning schedules. Gender differences in the development of PI (Spector *et al*. 1988, Bergstrom *et al*. 1996) and the implementation of pressure reduction mattresses (Bergstrom *et al*. 1996) have been also reported. Perhaps male participants in our study did not receive the assistance required to reposition themselves, or it could be that nurses interpreted participants reduced repositioning, as them lacking the motivation to move (Brown *et al*. 2007).

### Limitations

While we have used robust and systematic methods in this study, several limitations exist. The results presented in our study only relate to the participants at the research sites, so care is advised when interpreting these results. The frequency of repositioning was measured every 30 minutes and did not include any patient repositioning, which may have occurred in between the observation periods. This study only examined predictors of the frequency of repositioning and not the causal factors that might relate to the patient, nurses and the organization. A deeper examination of these factors was beyond the scope of this study; however, this is an area that warrants further investigation.

## Conclusion

In conclusion, this study found patients were more active than we might have thought, but they tend to adopt positions in bed that increase their risk of PI. Patients were repositioned less at night, than they were during the day; an issue compounded by reduced staffing. Older patients and males were repositioned less frequently, placing them at greater risk for PI development. Current PIP practice should be supplemented by the development of educational strategies that encourage a collaboration of nurses’ and patients in PIP.

### Relevance to clinical practice

Considered a cornerstone of PIP, the implementation of repositioning is an area requiring attention by clinicians, managers and researchers. Patients who are able, should be encouraged to participate in their PIP care. On admission to hospital, nurses should educate patients about PIP and the benefits of their participation in their care. Targeted and individualized PIP management should be incorporated into clinical practice, however, this can only be achieved with nurses support and concurrent organizational support.

## Conflict of interest

The authors report they have no conflict of interest.
